# Shaping Ability of Nickel-Titanium Rotary Instruments in Curved Root Canals

**Published:** 2013-05-01

**Authors:** Ali Talati, Saeed Moradi, Maryam Forghani, Ali Monajemzadeh

**Affiliations:** 1 Dental Research Center, Mashhad University of Medical Sciences, Mashhad, Iran; 2 Dental Materials Research Center, Mashhad University of Medical Sciences, Mashhad, Iran; 3 Department of Endodontics, Ahvaz University of Medical Sciences, Ahvaz, Iran

**Keywords:** Dental Instruments, NiTi Alloy, Root Canal, Root Canal Preparation

## Abstract

**Introduction:**

Disinfection and subsequent obturation of the root canal space require adequate mechanical enlargement of the canals. The purpose of this in vitro study was to compare the shaping ability of Mtwo, RaCe and Medin rotary instruments during the preparation of curved root canals.

**Materials and Methods:**

Sixty mesiobuccal root canals of mandibular molars with severe curvatures between 25-35^°^ and radius of 4-9 mm were randomly divided into three groups of 20 canals each. Using pre- and post-instrumentation radiographs, straightening of the canal and the apical transportation were determined with AutoCAD software. The data were analyzed using Chi square, analysis of variance, and post-hoc tests and the significance level was set at *P*<0.05.

**Results:**

Mtwo instruments maintained the canal curvature significantly better than Race and Medin instruments (*P*<0.05). There was significant difference between the rotary instruments for iatrogenic transportation of the major foramen (*P*>0.05).

**Conclusions:**

Under the conditions of this *in vitro* study, Mtwo instruments seemed superior to the two other rotary instruments.

## 1. Introduction

Mechanical root canal preparation is one of the most important stages in endodontic therapy that goes hand in hand with canal disinfection and subsequent obturation (1). The prepared root canal should have a continuously tapered funnel shape while maintaining the original canal curvature and apical foramen (2). These objectives are often difficult to achieve because of the highly variable root canal anatomy (3) and the design and physical characteristics of the instruments. During instrumentation, a number of procedural errors such as apical transportation, zip and ledge formation can occur along with loss of working length. Various instruments and root canal preparation techniques have been introduced to reduce these problems.

Rotary NiTi instruments are manufactured with various tapers that facilitate achievement of a continuously tapered funnel shape canal preparation. These instruments have been shown to be well suited for use in curved root canals (4, 5). Several methodologies were used to evaluate the flexibility and shaping ability of different NiTi instruments. Radiographic technique is a noninvasive method that successfully used by several investigators (6-8).

Mtwo (VDW, Munich, Germany), Race (FKGD entair, La-Chaux-de-fonds, Switzerland) and Medin (A.S., Czech Republic) are three NiTi rotary systems with good cleaning effectiveness (9-11).

Mtwo instruments have S-shaped cross-sectional design and a non-cutting safety tip. They are characterized by a positive rake angle with two cutting edges, which can effectively cut dentin. The pitch length increases from tip to shaft, eliminating the threading and binding in continuous rotation and reducing the transportation of debris towards the apex. Schafer et al. found that Mtwo instruments have good results in shaping ability/cleaning effectiveness (9).

RaCe instruments (Reamer with Alternating Cutting Edges) have a triangular cross-sectional design with sharp cutting edges. The exception is the 0.02 tapered size 20 files, which have a square cross-section. Schafer et al. found Race instruments removed debris effectively while maintaining the original outline form of the canal (10).

Medin instruments with various tapers have been introduced for root canal preparation. According to manufacturer, these files are designed to instrument difficult and curved root canals using “crown down” technique. Bidar et al. compared the cleaning efficiency of Mtwo, RaCe and Medin instruments and no significantly differences were found between groups in terms of smear layer removal (11).

However, little is known about the canal shaping ability of this NiTi rotary system. Therefore, the purpose of this in vitro study was to compare Mtwo, RaCe, and Medin rotary instruments ability to maintain or straighten curved root canals and too assess iatrogenic transportation of major apical foramen using radiographic technique.

## 2. Material and Methods

Sixty extracted, human mandibular first molars with intact root apices were selected for this in vitro study. The teeth were stored in 5.25% sodium hypochlorite for 1 h for cleaning purposes. Coronal access was prepared using a diamond bur (FG 801 Diatech, Heerbrug, Switzerland) and the canals were controlled for apical patency with a file of size 10.

Pre-instrumentation standardized radiographs were taken while the initial file of size 15 inserted into the mesiobuccal canal. The teeth were mounted in a radiographic mount made of silicon based impression material to produce a constant position. The X-ray cone (Siemens, Heliodent, Germany) was aligned perpendicular to the root canal. The exposure time was identical for all radiographs with a constant source-to-film and object-to-film distances. The same team member processed all films to increase consistency.

The canal curvature was determined according to Schneider (12), and the radius of curvature was calculated using the Schafer method (6). Only teeth with canal curvature between 25-35° and a radius of curvature ranged between 4.0 and 9.0 mm were included.

### 2.1. Root canal instrumentation

The teeth were randomly divided into three groups (n=20 each). Homogeneity of the groups with respect to the degree and radius of the curvature was evaluated using ANOVA. The working length was determined by measuring the length of the initial instrument at the apical foramen minus 1mm. The mesiobuccal canals were instrumented with Mtwo, Race or Medin. All root canal instrumentation were completed by one operator and all types of Rotary files were used with a 4:1 reduction hand piece (WD-60 EM; W&H) powered by a torque-limited electric motor (Endo IT; VDW). The rotational speed and torque limit for the rotary files were chosen according to the manufacturer’s instructions. The canals were irrigated with 5 mL of a 2.5% NaOCl solution after each instrument and 5 mL of normal saline was used as final rinse.

In Group 1, Mtwo files were used with single-length technique according to the manufacturer’s instructions using a gentle in-and-out motion. Four instruments were used, Mtwo 10.04, 15.05, 20.06, and 25.06.

In Group 2, RaCe files were used in a crown down technique according to the manufacturer’s instructions. The instrumentation sequence was RaCe 40.08, 35.08, 30.06, 25.04, and 25.02.

In Group 3, canals were prepared with the Medin system. These files were also used in a crown down method according to the manufacture’s guidelines with this sequence: w1 (10.04), w2 (15.05), w3 (20.06), and w4 (25.06).

In all groups the final apical preparation diameter was size 25.

### 2.2. Evaluation and Statistical Analysis

After the canal instrumentation, the canal curvatures were determined again on the basis of a radiograph with the final root canal instrument inserted into the canal. The straightening of the canals was determined as the difference between canal curvature prior to and after canal instrumentation. To assess the transportation of major apical foramen, two periapical radiographs (including the initial and final instrument) were superimposed with AutoCAD software. When the files were not superimposed at the apical area (ending at the apex), transportation was assumed.

Assessment of the changes in the canal curvature and apical transportation was carried out by an examiner who was blind to all experimental groups.

The changes in canal curvature were analyzed statistically using the analysis of variance (ANOVA), and post-hoc student Newman-Keuls tests at a significance level of P<0.05. The data established for the apical transportation were subjected to the Chi square tests. P-values were compared to the P=0.05 level.

## 3. Results

Experimental groups were well balanced according to Mean and standard deviation (SD) of canal curvature before and after canal instrumentation (Table 1).

**Table 1.** Degree of straightening of root canal curvature

	Mean (SD) of canal curvature	
Groups	preoperative	postoperative
**Mtwo*(n=20)***	29.97±2.55	28.24±2.40
**Race*(n=20)***	30.03±2.56	26.62±1.35
**Medin*(n=20)***	29.99±2.56	26.47±1.23

The use of Mtwo instruments resulted in significantly less canal curvature changes (mean±SD: 1.735±0.566) compared with RaCe (3.405±1.729) and Medin (3.515±1.682) (*P*<0.05). No significant difference was obtained between RaCe and Medin.

The results for apical transportation were similar and no significant difference was found between three experimental groups (*X*^2^=2.1; *P*=0.340) (Table 2).

**Table 2.** Comparison of apical transportation between 3 groups

	Transported	
Groups	N	%
**Mtwo***(n=20)*	0	0
**Race***(n=20)*	2	10
**Medin***(n=20)*	2	10

## 4. Discussion

Sixty extracted, human molars were selected for this in vitro study. Though the use of extracted teeth (due to complex root anatomy and the variability in dentin structure) compromised standardization of the experimental groups, natural teeth provide conditions close to clinical situation. Simulated canals in resin blocks are a method to standardize conditions (13). Bertrand et al. demonstrated that the use of resin blocks may not represent clinical conditions because it doesn’t reflect dentinal structure and rigidity (14). Resin block is not ideal for studying rotary files due to different structures of resin and dentin (15), and the generated heat may sometimes soften the resin so that instrument’s blade may bind and break (16). In the present study, the experimental groups were homogenous with regard to the angle and the radius of canal curvature, as well as using mesiobuccal canals of mandibular molars, and the use of single operator for canal instrumentation.

Teeth with canal curvature between 25-35^°^ and a radius of curvature ranged between 4.0 and 9.0 mm were considered as severely curved canals. This inclusion criteria was similar to previous studies (8, 10, 17).

During the filing process, the Endo IT motor was used to control the torque speed (18). According to one previous study (19), Endo IT prevents file breakage by counter-rotating the file.

Several techniques can be used to evaluate the shaping ability of NiTi instruments, such as serial sectioning technique, micro-computed tomography (micro-CT), and radiographic technique. Each of these methods has distinct advantages and disadvantages. For example, the serial sectioning technique is invasive and restricted to predetermined levels (20) and results in unknown tissue changes and loss of material (21). Micro-CT is not cost-effective. The radiographic technique is noninvasive but only is used to record two-dimensional changes (22). However, Katz and Tomase demonstrated that apical transport displays the greatest changes in the mesiodistal dimension (23). Therefore, the radiographic method reported by Schafer et al. (9) was used in our study to determine canal transportation.

The most important aspects in evaluating the shaping ability of instruments are preservation of the original canal path and a central position of the file (17, 24). In the present study, the use of Mtwo instruments resulted in significantly less canal curvature changes compared with RaCe and Medin. The good shaping ability of Mtwo agrees with simulated and in natural tooth canal studies (9, 25, 26). It might be due to double cutting-edge geometry and smaller cross-section area; which increases their flexibility (25).

Several previous studies reported the appropriateness of RaCe rotary files (10, 27, 28) for maintaining and shaping curved root canals. However, our study demonstrated more canals straightening with RaCe instruments compared to Mtwo. Ozgur Uyanik et al. have investigated root canal preparation with Hero Shaper, ProTaper, and RaCe. They found that RaCe files significantly transported the canals at the coronal level. The authors mentioned that this finding may be a result of the stainless-steel nature of the first precoronal flaring files of the RaCe system (29). Al-Sudani et al. also reported that the RaCe system significantly showed more transportation compared to the ProFile and K3 systems (30). Moradi *et al.* compared centering ability of three rotary systems in curved root canals and reported that best centering ability was obtained by Mtwo instruments compare to Race and Medin instruments (31).

Our results showed similar degree of transportation of apical foramen in all groups. In 2 specimens, apical transport occurred following instrumentation with Medin and RaCe. However, such differences were not statistically significant and may therefore be irrelevant for clinical practice. Previous study using radiography revealed that most often reduction in the canal curvature is not associated with apparent apical transportation (32). This finding was confirmed by our results.

## 5. Conclusion

In the present *in vitro* study Mtwo NiTi rotary system was superior to Race and Medin NiTi rotary instruments in respect of maintaining canal curvature.
